# Tobacco is “our industry and we must support it”: Exploring the potential implications of Zimbabwe’s accession to the Framework Convention on Tobacco Control

**DOI:** 10.1186/s12992-015-0139-3

**Published:** 2016-01-11

**Authors:** E. Anne Lown, Patricia A. McDaniel, Ruth E. Malone

**Affiliations:** Department of Social and Behavioral Sciences, School of Nursing, University of California, San Francisco, CA 94143-0612 USA

**Keywords:** Tobacco industry, Agriculture, Zimbabwe, Developing countries, World Health Organization, FCTC, Global health, Humans, Smoking*/prevention & control, International cooperation

## Abstract

**Background:**

Zimbabwe is the largest tobacco producer in Africa. Despite expressing opposition in the past, Zimbabwe recently acceded to the World Health Organization’s Framework Convention on Tobacco Control (FCTC). We explored why Zimbabwe acceded to the FCTC and the potential implications for tobacco control within Zimbabwe and globally.

**Methods:**

We conducted a qualitative archival case study based on 542 documents collected from 1) the Truth Tobacco Industry Documents; 2) media indexed in the Lexis-Nexis media database; 3) the websites for tobacco growers’ associations, tobacco control groups, and international agencies; 4) FCTC reports and Framework Convention Alliance newsletters; 5) Zimbabwe’s legal codes; and 6) the peer reviewed scientific literature related to tobacco growing.

**Results:**

Zimbabwe has a long history of tobacco growing. There are currently over 90,000 tobacco farmers, and tobacco growing is prioritized, despite widespread food insecurity and environmental degradation. Zimbabwean government officials have been outspoken FCTC critics; but recently joined the accord to better protect Zimbabwe’s tobacco growing interests. FCTC membership obligates nations to implement a variety of tobacco control measures; Zimbabwe has implemented several measures aimed at reducing tobacco demand, but fewer aimed at reducing tobacco supply or protecting the environment. Zimbabwe joins the FCTC amid increased efforts to protect FCTC proceedings from industry interference, to adopt recommendations for alternative crops and livelihoods and reduce environmental damage.

**Conclusion:**

Zimbabwe’s decision to accede to the FCTC does not appear to represent a softening of its historical opposition to the treaty. Thus, its status as a Party creates opportunities for it to undermine ongoing efforts to implement and strengthen the treaty. At the same time, however, Zimbabwe’s accession could provide much needed international support for Zimbabwe’s civic organizations and its Ministry of Health to develop stronger tobacco control measures. How Zimbabwe’s participation impacts the work of the FCTC as a whole may ultimately depend on the allegiances of its delegates, and the effectiveness of FCTC measures to limit tobacco industry interference and enforce compliance with FCTC measures.

## Background

While tobacco use is leveling off in high-income countries such as the US and Britain, the tobacco industry is aggressively marketing to low and middle-income nations [1]. Nonetheless, many African countries still have relatively low rates of tobacco use ([2] pp. 268) suggesting that the tobacco epidemic that killed 100 million in the 20th century, mostly in higher income nations, could still be averted there. Tobacco use is distinguished from many other health problems by the presence of an aggressive, transnational tobacco industry whose goals are fundamentally incompatible with public health [3]. Like other industries, the tobacco industry not only seeks to promote use of its products and expand into new markets, but also seeks to weaken strong tobacco control policies and undermine public health advocacy efforts [4–8].

African nations played an integral role in developing and establishing a strong policy response to the tobacco problem, the United Nations World Health Organization’s Framework Convention on Tobacco Control (WHO-FCTC) [9, 10]. The FCTC treaty entered into force in 2005 with 40 signatories. In contrast to most of its African neighbors, Zimbabwe, one of the largest tobacco producers in Africa and the world, [11] was opposed to the FCTC’s creation [12, 13]. However, on December 4, 2014, Zimbabwe deposited a signed version of the FCTC treaty to the United Nations and on March 4, 2015 Zimbabwe officially became the 180th Party to the accord [14–16].

It is unknown what positions Zimbabwe will take as a Party to the FCTC. If Zimbabwe uses its influence to obstruct, delay, or diminish FCTC provisions related to tobacco growing, marketing and distribution, its accession would represent a setback for global tobacco control efforts. Adopting the role of obstructionist would also place Zimbabwe at odds with many of its African neighbors, who continue to be actively engaged in implementing the FCTC. However, as a negotiating Party, Zimbabwe may also find itself forced to compromise in ways that ultimately benefit tobacco control efforts within the country and within the region.

This paper describes the role that tobacco plays in Zimbabwe’s economy, and why the country acceded to the FCTC. Next, it examines the obligations and privileges associated with FCTC membership, and concludes by considering the possible implications of Zimbabwe’s accession to the FCTC for both the international tobacco control movement and for tobacco control within the country.

## Methods

In this archival qualitative study we examined data from multiple sources. First, to identify tobacco industry influence in Zimbabwe, the first and second authors searched the Truth Tobacco Industry Documents (TTID) (https://industrydocuments.library.ucsf.edu/tobacco/). The TTID contains over 14 million internal tobacco industry documents released as part of the 1998 Master Settlement Agreement between the attorneys general of 46 U.S. states and the tobacco industry [17]. To locate documents, we used search terms for tobacco growers’ associations active in Zimbabwe (e.g., “International Tobacco Growers Association,” “Zimbabwe Tobacco Association”), as well as tobacco control activities (e.g., “World No Tobacco Day”), and tobacco industry activity (e.g., “British American Tobacco-Zimbabwe,” “Savanna Tobacco,” “Courtesy of Choice Campaign”) in Zimbabwe. We retrieved and screened over 5,000 documents; after eliminating duplicates and irrelevant materials, we examined in more detail 45 TTIDs.

Because the bulk of tobacco industry documents we found were dated from 1981 to 2000, the first author also searched the Lexis Nexis database for media accounts of the current situation in Zimbabwe. LexisNexis indexes over 26,000 media sources, including local, national and international newspapers, magazines, trade journals, and radio and television broadcast transcripts. She used search terms related to Zimbabwe and tobacco (e.g., “Zimbabwe” AND “tobacco” OR “cigarette” OR “smoking” OR “tobacco control” OR “FCTC”) and to political, health, and economic dynamics in Zimbabwe (e.g., “Zimbabwe” AND “sustainable development goals” OR “world tobacco day” OR “deforestation” OR “environment” OR “child labour/labor” OR “tobacco exports” OR “land reform”). She found 168 relevant media items; most were newspaper or magazine articles or transcripts of radio or television broadcasts from news sources in Zimbabwe, the UK, the US, and China.

For the most current information on the activities of tobacco growers’ associations and tobacco manufacturers in Zimbabwe, the first and second authors examined their websites (e.g., tobaccoleaf.org, fctobacco.com, bat.com, savanatobacco.com). To understand the history and process of the WHO-FCTC, the first author examined online WHO-FCTC reports (e.g., “Conference of the Parties to the WHO FCTC, No 5, Friday, 17 October 2014”) and Framework Convention Alliance (FCA) newsletters and reports. To understand international tobacco issues the first and second authors examined various websites (e.g., the United Nations Development Program), (N = 94) reports (e.g., Food and Agriculture Organization or World Bank) (N = 98), proceedings (e.g., All Africa Conference on Tobacco Control) (N = 4), and books or book chapters (e.g., *Tobacco Control in Africa: People, Politics and Policies*) (N = 7). The authors also examined the text of Zimbabwe’s tobacco control law (e.g., Statutory Instrument 264 of 2002). Finally, in order to place our findings in context, the first and second authors examined the peer reviewed scientific literature related to tobacco growing, Africa and/or the FCTC (N = 122). The total number of documents that served as the basis for our analysis was 542.

We followed an approach consistent with a qualitative case study [18]. All relevant documents were downloaded into an Endnote database, notes were added, key words were assigned, and key pieces of information were highlighted (e.g., policies on land use and tobacco growing, comments from Health Ministers about FCTC or tobacco control measures). The documents were organized chronologically and by topic (e.g., FCTC-related statements made over time by Zimbabwe government officials). For validation purposes we asked two experts involved in FCTC negotiations to review the penultimate draft of our report.

## Results

### Tobacco’s role in Zimbabwe’s economy

The Zimbabwean government regards tobacco as the “lifeblood of Zimbabwe’s economy” [13]. In 2012 the country was the top tobacco-producing nation in Africa, and in 2013 it was the sixth largest tobacco producer in the world [11]. According to Zimbabwe’s Tobacco Industry and Marketing Board, 98 % of Zimbabwe’s tobacco is exported, making it the country’s largest foreign currency generator (accounting for 10–43 % of the country’s gross domestic product) [19, 20]. Zimbabwe receives a higher percent of government revenue from tobacco leaf than any other country in the world, except Malawi [21], partly due to a levy system that taxes both growers and buyers [22].

Tobacco is a major source of employment: there are over 90,000 small scale tobacco farmers in Zimbabwe [19, 23], supported by a robust tobacco growing infrastructure. For example, banks dedicate significant revenue for loans to tobacco farmers [24], though small-scale farmers have fewer loan options [25]), land (taken in 2000 from white farmers) is preferentially given to black tobacco farmers by the government [26–28], tobacco companies offer loans for seeds and fertilizer [19, 24], and tobacco farmers receive cash payments upon delivering their crop [29]. As a result, despite widespread hunger in Zimbabwe, farmers are more likely to grow tobacco than grain [19, 24].

Zimbabwe’s Ministry of Agriculture and Ministry of Industry and Commerce oversee tobacco industry-related organizations such as The Tobacco Research Board, the Tobacco Industry and Marketing Board, and the Boka Tobacco Auction Floors. Two independent groups that claim to represent tobacco farmers’ interests, the Zimbabwe Tobacco Association (ZTA) (and its offshoot, the Farmers Development Trust, recipient of several US$100,000 grants from Phillip Morris [30]), and the International Tobacco Growers Association (ITGA), are influential in Zimbabwe [31, 32]. The ZTA was founded in 1928 (originally as the Rhodesian Tobacco Association) to “promote and support research and training to ensure the continued development and expansion of the flue-cured tobacco growing industry” [32]. In 1984, the ZTA and representatives of five other tobacco-growing nations founded the ITGA with funding from transnational tobacco companies [33–35]. Previous research has exposed the ITGA as a public relations vehicle for transnational tobacco companies, providing a “human face” and a “grass roots voice” to articulate the positions of tobacco manufacturers [35, 36]. Both groups have publicly criticized the FCTC [12, 13, 32, 34, 37] and both have tried to influence FCTC proceedings [36, 38, 39].

While industry, government, and tobacco grower organizations in Zimbabwe work together to support tobacco growing, their interests and positions sometimes conflict. For example, the ZTA opposed government levies on growers and a land redistribution program in the late 1990s, which resulted in many highly productive white tobacco farmers fleeing to Zambia, South Africa, and Mozambique [40, 41] (where tobacco growing subsequently increased) [11].

Zimbabwe’s economy is near collapse due to its US$11 billion debt [42], made worse by continuing corruption [42–44], high unemployment (80–85 %) [42, 45, 46], widespread hunger [47], succession battles [42, 44, 48–51], and a 2014 law that requires all companies to hand over 51 % of shares to black Zimbabweans [52]. In the face of economic strains, tobacco growing is likely to continue to be a major income generator for the government.

### Zimbabwe’s accession to the FCTC

In the past, Zimbabwe government officials and growers’ organizations have been outspoken supporters of tobacco growing and critics of the FCTC [31, 53, 54]. For example, Zimbabwe’s President Robert Mugabe expressed support early in his presidency for the tobacco industry saying: “If we sell it, we must grow it as well. It is our industry and we must support it” [31]. In 2000, at the FCTC public hearings in Geneva, the ZTA criticized the work of the FCTC as a “crusading task of drawing up a global tobacco prohibition accord, to be legally imposed upon governments” [12]. (The FCTC does not, in fact, ban tobacco growing [55]). Both the ZTA and the ITGA developed a briefing on the FCTC describing it as a “thoroughly bad and damaging international treaty” which represented an “an attack on [Zimbabwe’s] national sovereignty” [32].

By 2010, as the FCTC garnered increasing international support, Zimbabwe appeared to re-assess the value of its outsider status. Joseph Made, Zimbabwe’s Minister of Agriculture and close ally of President Mugabe, argued that it was time to join the FCTC because the country’s outsider status made it more difficult to protect its tobacco interests and it needed to work in concert with other countries [13]. In 2013, Gavin Foster, ZTA president, appealed to the Zimbabwean government to sign the FCTC accord “so that we can defend not only our tobacco growing industry but that of the entire continent under threat. Lets [sic] us stop non producing and non tobacco [sic] dependent countries; countries and organisations with hidden agendas deciding the future of our industry and livelihoods” [34]!

### The need for tobacco control in Zimbabwe

While daily smoking prevalence among women in Zimbabwe is relatively low (5 %), men’s smoking prevalence is much higher (33 %) [56], reflecting the growing popularity of smoking among African men [57]. In Africa, civil society organizations have played a key role in spurring government tobacco control [58], but in Zimbabwe only one civic tobacco control organization, the Zimbabwe Framework for Tobacco Control Trust, is apparently active. It applied to attend a recent FCTC meeting but was turned away as it has no website, no identifiable members, and scant media coverage [19, 59]. There is a demonstrable need for tobacco control in Zimbabwe.

### FCTC Membership: Obligations and Privileges

In addition to several general obligations, the FCTC requires members to adopt and implement measures that address tobacco control in three domains -- tobacco demand reduction, tobacco supply reduction, and protecting the environment -- as spelled out in the 18 primary articles that comprise the treaty. Tables 1, 2, 3 and 4 outline how Zimbabwe’s current tobacco control policies and practices measure up against FCTC articles.

**Table 1:** Zimbabwe’s 2015 tobacco control policies in relation to FCTC general obligations

FCTC Article [60]	Zimbabwe’s tobacco control policies	Zimbabwe’s practice
Article 5: Develop and implement tobacco control measures; finance and coordinate the work nationally.	• One law, Statutory Instrument 264 of 2002, outlines smoke-free premises, no smoking signs, tobacco and children, product ingredients disclosures, promotion of tobacco products, and importing tobacco.	• The government rationale for joining the FCTC was: “[w]e cannot fight from outside and win [13].”-- Minister of Agriculture (2010) • “We will grow [tobacco] for those who want to smoke it. You should listen to what your doctor says. But if you over smoke, don’t blame us [64].”-- President Robert Mugabe (April, 2015) • The Minister of Agriculture-Joseph Made described an unusual unity between black and white farmers in order to save tobacco-Zimbabwe’s dominant cash crop. “There are no differences between us on this one,” he says. “Everyone is working together….[13]” (2010) • Economic problems and corruption [42–44] slow tobacco control implementation and coordination.
Article 5.3: Protect tobacco control policies from commercial and other vested interests of the tobacco industry.	• No laws protect tobacco control policies from tobacco industry interests.	• Media reports credit nepotism for government’s failure to act on Zimbabwe’s Savanna Tobacco Company smuggling charges. Savanna Tobacco is owned by Mugabe’s relative [75, 81]. • “There is need for all of us to be aware of the tobacco industry’s activities to undermine tobacco control efforts through advertising, promotion and sponsorship which lure you into believing that tobacco is good…We will not tolerate any interference from the tobacco industry as we go about our duty of forming and enforcing laws that are good for the health of our people [110].”-- Minister of Health Madzorera (2013) • Zimbabwe government ministry accepted $527,000 from BAT to support small tobacco farmers [65].

**Table 2:** Zimbabwe’s 2015 tobacco control policies in relation to FCTC measures to reduce demand for tobacco

FCTC Article [60]	Zimbabwe’s tobacco control policies & activities	Zimbabwe’s practice
Article 6: Price and tax measures to reduce the demand for tobacco	• Tax is 45 % of the retail price of cigarettes. [111]	• Tobacco growers and leaf buyers are also taxed [22].
Article 8: Protection from exposure to tobacco smoke	• 3 smoke-free public places: health care institutions; non-University educational facilities; and public transport [68]. • Fine of Z$500, imprisonment or both for violators [112].	• No/scant enforcement for passive smoking laws-Ministry of Health [113].
Article 9: Regulation of the contents of tobacco products	• No regulations identified.	
Article 10: Regulation of tobacco product disclosures	• Statutory Instrument 264, sec 7 • “Every tobacco product shall bear accurate information on the percentage of the tar and nicotine content and any other ingredients…visible on the package [61].”	
Article 11: Packaging and labeling of tobacco products	• Brand descriptors (e.g.,“light,” “low tar”) allowed [9, 56]. • 20 % of cigarette package must be covered by health warning [9, 56, 63]. • Packaging must contain one of three health warnings [61].	• Inexpensive, single stick cigarette sales are lucrative marketing strategy in Africa [114, 115] aimed at low income, low education, and young smokers [114]. • Smuggled single stick packages create tobacco control challenges for surrounding countries [74] • BAT-Z accused of selling cigarettes without Zimbabwe’s prescribed warnings [116].
Article 12: Education, communication, training and public awareness of tobacco control issues	• No mass education campaigns implemented between 2012-2014 [56, 68]. • World No Tobacco Day has been celebrated in Zimbabwe since 2013 [117, 118].	• Government officials minimize risk of tobacco use [34, 53]. • No whole population media anti-smoking messages [63, 111].
Article 13: Comprehensive ban on tobacco advertising, promotion and sponsorship	• No direct or indirect bans on tobacco advertising, promotion or sponsorship [56, 68], (except,visual entertainment) [9, 56]. • Promotional events are only allowed for adults [61]. • No bans on free cigarette distribution, promotional discounts, sponsored events, or corporate social responsibility activities [56].	• Savanna Tobacco Co. sponsored Miss Zimbabwe contest [70], Zimbabwe’s signature musician, Oliver Mtukudzi [71], local soccer teams [72, 73], and proposed $10 million donation for Harare stadium to be named after its Pacific brand [69]. • 63–77 % of youth exposed to tobacco advertising and 69–86 % exposed to brand names at sports events [82]. • Tobacco marketing in Zimbabwe--more aggressive than in high income countries [119].
Article 14: Demand reduction measures concerning tobacco dependence and cessation.	• Nicotine Replacement Therapy (sold with prescription) and/or some smoking cessation services available; costs not covered [68]. No national quitline [111].

**Table 3:** Zimbabwe’s 2015 tobacco control policies in relation to FCTC measures to reduce supply for tobacco

FCTC Article [60]	Zimbabwe’s tobacco control policies & activities	Zimbabwe’s practice
Article 15: Eliminate illicit trade in tobacco products.	• No tax stamps, local-language cigarette pack warnings, aggressive enforcement, or penalties in place [120].	• Cigarette smuggling by STC and BAT-Z widely reported in media [75–81].
Article 16: Prohibit sales to and by minors	• Sales of tobacco to under age 18 is prohibited [61]. • Enforcement information unavailable.	• 12 % of youth-current smokers [56] • 28–49 % bought cigarettes in a store [82].
Article 17: Provision of support for economically viable alternate activities	• No government sponsored programs to promote alternative crops.	• Ronald Watts, Zambian agricultural consultant, listed 53 possible alternative crops that could be developed in the region (1993) [84, 121]. • “There are no sustainable, economic [ally] viable alternate crops to tobacco [34].” ZTA chairman (2014) • Government and industry officials say- no economically viable alternative crops exist to replace tobacco [34, 83]. • Incentives for tobacco growing remain strong with increasing acreage devoted to it [122, 123]. • Local media favor stories of successful tobacco growing [85, 86].

**Table 4:** Zimbabwe’s 2015 tobacco control policies in relation to FCTC measure to protect the environment

FCTC Article [60]	Zimbabwe’s tobacco control policies & activities	Zimbabwe’s practice
Article 18: Due regard for the protection of the environment and the health of persons in respect to tobacco cultivation and manufacture	• Section 4 of Zimbabwe’s Environmental Management Act (Chapter 20:27), 2002, describes 4 general environmental rights: 1) Clean healthy environment; 2) Access to environmental information; 3) Environment to be protected for benefit of present and future generations; 4) Right to promote policies to end pollution and environmental degradation, and support sustainable management of resources [124]. • Statutory Instrument 116 of 2012 regulates use and trade of firewood and timber [125].	• Deforestation is widespread [23, 87–92] threatening to denude the country by 2016 [23, 88, 91, 93, 126]. • Zimbabwe is among top 10 countries for largest forest cover loss between 1990 and 2010 [127]. • Child labor on tobacco farms exposes children to harmful pesticides and fertilizers and prevents school attendance [128, 129]. • Tobacco pickers risk nicotine poisoning [130, 131]. • Tobacco industry sponsors Sustainable Afforestation tree planting program [91, 132].

General obligations for FCTC Parties as outlined in Article 5 require member countries to actively engage in tobacco control work, financing and coordinating the work on a national level (Table 1) [60]. Zimbabwe has few tobacco control measures and one national tobacco control law, Statutory Instrument 264 of 2002 [61]. In 2009 it had a national tobacco control agency (with one employee), and no budget for tobacco control activities [62]. In 2014 Zimbabwe developed national tobacco control objectives [63]. President Mugabe continues to publicly express support for tobacco growing [64].

FCTC Article 5.3 specifies that tobacco control measures should be protected from commercial or other vested interests of the tobacco industry [60]. Just four months after acceding to the FCTC, a Zimbabwean government ministry signed a Memorandum of Understanding accepting a donation of $527,000 from the British American Tobacco (BAT) to enhance tobacco production by providing training, building tobacco curing barns, and improving access to credit for youth, women, and disabled small farmers [65–67].

Tobacco control activities to reduce demand (Articles 6-16) in Zimbabwe have been minimal (Table 2). Health warnings are required on cigarette packages [56], there are some designated smoke-free settings [68], and cigarettes are taxed at 60 % of the retail price [68]. Beyond that, there are few other regulations. Cigarettes are widely advertised [56, 68], sponsorships by Savanna Tobacco, a local Zimbabwean company, are common [69–73], and single stick cigarette sales are popular to market smoking to the poor [74]. There are no mass education anti-smoking programs [56, 68], and government and tobacco industry officials have publicly minimized the dangers of smoking [34, 53]. For example, in 1994, at a national congress meeting, President Mugabe minimized the risk of tobacco use when he said, “I think WHO has its priorities wrong. Why can they not be more fair with tobacco and start with alcohol” [53]. At the 2013 World Tobacco Day ZTA president Gavin Foster said, “The impact of tobacco on the health of individuals whether on us growers, our workers and families, processors, manufacturers, our end user the smoker and the general populace ranks very low when ranked against the World Health Organisation’s deadliest causes of death and diseases such as heart disease, strokes, lower respiratory diseases, HIV, and other NCDs” [34].

Tobacco control activities aimed at reducing the supply of tobacco (Articles 15-17) have been even less vigorously pursued. There are widespread reports of cigarette smuggling to surrounding countries [74–81]. While there are laws to prevent tobacco sales to minors, [61] young urban and rural dwellers report easy access (Table 3) [82]. Article 17 is particularly challenging for Zimbabwe since it involves the “provision of support for economically viable alternative activities” to tobacco growing. Both government and industry officials claim that there are no economically viable alternative crops to replace tobacco, [34, 83] despite evidence to the contrary [84]. Local media reports continue to emphasize the advantages of growing tobacco [85, 86].

Finally, tobacco control efforts aimed at the “protection of the environment and health of persons” (Article 18) represent another hurdle (Table 4) [60]. Deforestation is a particularly significant problem for Zimbabwe [23, 87–93], since flue-cured tobacco requires heat to process the leaves, and wood is used as a fuel supply [87].

A key privilege of joining the FCTC is becoming a voting member at Conference of the Party (COP) meetings [14]. COP is the governing body of the FCTC and is comprised of its 180 Parties [60]. Parties outline, adopt, and amend policies, make decisions on implementation, and discuss compliance with FCTC articles [94]. FCTC rules stipulate that COP meets in regular sessions biennially and that further work happens in regional meetings and designated working groups between COP meetings [95]. Membership in working and regional groups is voluntary. Decision-making is by consensus among Parties (observers can comment), with the caveat that a three-quarter majority rule is acceptable for substantive matters or a simple majority rule for procedural matters [95]. In past practice, consensus has dominated decision-making. Given the preference for consensus and voluntary membership in regional and working groups, there are opportunities for a Party to influence or obstruct FCTC provisions that it finds objectionable. Moreover, as a member of COP, Zimbabwe will be party to negotiations and have access to documents and conversations that would likely be of interest to the tobacco industry.

In acceding to the FCTC, Zimbabwe has joined a club to which 43 of 47 African nations belong. (Only Eritrea, Malawi, and South Sudan have not ratified the FCTC. Mozambique has signed but not ratified the treaty [16, 96]). African regional unity was key to negotiating a strong treaty, with African nations (both tobacco producing and non-producing) voting as a bloc and making alliances with other regional blocs to advance their tobacco control agenda [9, 10]. Thus, at COP meetings, Zimbabwe may contend with pressure to advance not simply its own interests, but regional interests as well [96].

Future COP meetings are likely to discuss two issues of particular significance for Zimbabwe: strengthening Article 5.3, and reviewing progress on recommendations for implementing Articles 17 and 18. Article 5.3 specifies that tobacco control measures should be protected from commercial or other vested interests of the tobacco industry [60]. Recent COPs have accelerated efforts to comply with Article 5.3 [60] which was first adopted at COP3 [97]. At COP5, Parties requested further discussion about the large number of industry representatives among the public attendees at COP meetings. At COP6, discussion centered on the need for more rigorous and advance vetting of the public (including the media) at future COP meetings and the exclusion of observers at the current meeting, where all Party delegates (not just observers, as before) might be required to file a declaration denying “any form of real, perceived, or potential conflict of interest with the tobacco industry” ([98, 99], pp. 31-32).

Further discussion on minimizing industry influence and accelerating Article 5.3 implementation is likely to occur at COP7 scheduled to take place in New Delhi in late 2016. Specific language to strengthen Article 5.3 could result in the exclusion of media, observers, or even individual delegates from COP meetings; the mandatory use of new tools to track implementation of Article 5.3; increased transparency regarding industry involvement in Party countries; and the elimination of conflicts of interest for government officials and employees [100].

COP has been working on how best to implement Articles 17 and 18 since 2006. (Article 17 was included in the FCTC to protect tobacco farmers by addressing the possible economic consequences of effective tobacco control policies that could reduce demand for their crop [101].) At COP6 in 2014, an Articles 17/18 working group submitted its report. Although it was 8 years in the making, COP6 decided that the report needed further refinement. Parties quickly established an informal group to provide a brief set of nine recommendations drawn from the report ([99], p 16) COP6 adopted the policy options and recommendations in the full working group report despite divergent views [102] and requested that the Convention Secretariat implement the nine recommendations ([99], p. 79). The working group’s mandate was not renewed [99]. At COP7 (New Delhi, 2016), the Secretariat will provide a progress report on the implementation of the recommendations [99]. Issues related to planting alternative crops and reducing environmental and health damage from tobacco growing are contentious and complex and any proposed solutions are likely to pose challenges for Zimbabwe, now officially involved in future discussions.

## Discussion

Zimbabwe’s current economic hardship, its robust tobacco growing and distribution infrastructure, and continued world demand for tobacco suggest that the government will continue to prioritize tobacco production in the absence of incentives to do otherwise. Its recent decision to accede to the FCTC does not appear to represent a softening of its historical opposition to the treaty, but rather a strategic move to better protect and defend its tobacco interests in a world with a growing commitment to tobacco control.

Zimbabwe is not the first tobacco-dependent nation to sign on to the FCTC. Brazil, despite its status as one of the world’s top tobacco producers, was instrumental in the creation of the FCTC and has been successful in reducing tobacco use by 50 % [103, 104]. Like Zimbabwe, most of its tobacco growing revenue came from exports. Since tobacco control measures that target internal tobacco use cause little conflict with export profits, Brazil was able to meet many of its FCTC obligations [105]. South Africa and Zambia, despite having strong tobacco growing industries, have also been able to advance tobacco control [9, 106].

These examples suggest that Zimbabwe may also be able to implement tobacco control at home [105]. As a member of the FCTC, Zimbabwe’s internal tobacco control organizations and the Ministry of Health will now have international support to develop and promote stronger tobacco control measures [19]. Vigorous tracking of compliance with FCTC measures by the FCTC secretariat and public consequences for failure to meet obligations would further support tobacco control efforts in Zimbabwe [107].

Of concern is Zimbabwe’s recent acceptance of a financial donation from BAT just months after acceding to the FCTC. This action could jeopardize Zimbabwe’s participation in FCTC activities especially if there is stricter FCTC monitoring of compliance with Article 5.3 for “real, perceived, or potential conflict of interests” [99]. Acceptance of the BAT donation is a flagrant act of non-compliance with Articles 5.3, 17, and 18 [67].

Given these alliances Zimbabwe may undermine efforts to implement and strengthen the FCTC. For instance, its delegates could act as the eyes and the ears of the tobacco industry, reporting back on delegates’ activities and sharing draft documents. This occurred previously when a Brazilian delegate who made numerous calls to a Brazilian subsidiary of BAT during COP4 meetings [108]. The inclusion of delegates with tobacco industry agendas within FCTC does occur (although it has been declining), and there are no current clear mechanisms to prevent this until a formal vetting process for delegates is in place.

Zimbabwe’s status as a Party also offers it the opportunity to influence ongoing discussions about Articles 17 and 18. The FCTC has been slow to agree on a coherent and effective policy around supporting economically viable alternatives to tobacco growing and protecting the environment. Although the working group devoted to these issues no longer exists, discussions about alternative crops are likely to remain active, particularly if demand for tobacco declines. In the interim, Zimbabwe may be dismissive of many of the Articles 17/18 recommendations. Given COP’s preference for consensus-based decision-making, it may require little effort by Zimbabwe to further stall progress. Zimbabwe could be aided in this endeavor by its natural alliances with other tobacco growers in the region – particularly in those countries to which white Zimbabwean tobacco farmers emigrated. However, the strong bloc of unified African countries that have a history of support for the FCTC may also hold Zimbabwe in check. Some of Zimbabwe’s neighbors have much stronger tobacco control (e.g., Zambia). Zimbabwe’s accession to the FCTC may be welcome in the region since membership will provide an obligation for Zimbabwe to take measures to reduce smuggling.

## Conclusion

By 2025, much of Africa faces a worsening tobacco epidemic among men [109]. Implementing the FCTC’s demand reduction measures has the potential to reverse this outcome. By its own admission, Zimbabwe joined the FCTC as a Party in order to defend its tobacco growing interests, but its status as a Party may open doors for tobacco control. Zimbabwe’s participation in the FCTC as a whole, and progress on Articles 5.3, 17, and 18 in particular, may ultimately depend on allegiances that exist between Zimbabwe’s delegates and 1) the tobacco industry, 2) the Zimbabwean government, and 3) Parties within the African region, as well as vigorous FCTC monitoring for compliance. To encourage Zimbabwe to be a productive member of the FCTC, it will be necessary to increase pressure on Parties to resist tobacco industry interference and comply with treaty obligations.

## Abbreviations

BAT-Z: British American Tobacco Zimbabwe
COP: Conference of Parties
FCA: Framework Convention Alliance
ITGA: International Tobacco Growers Association
WHO FCTC: World Health Organization Framework Convention on Tobacco Control
ZTA: Zimbabwe Tobacco Association
